# Translational Science 22 conference proceedings

**DOI:** 10.1017/cts.2022.427

**Published:** 2022-07-12

**Authors:** Elina E. Pliakos, Laneisha K. Tague, Roy E. Poblete

**Affiliations:** 1 Department of Medicine, University of Pennsylvania, Philadelphia, PA, USA; 2 Department of Medicine, Washington University, St Louis, MO, USA; 3 Department of Neurology, Keck School of Medicine, University of Southern California, Los Angeles, CA, USA

**Keywords:** Translational science, health justice, public health emergency, community engagement

## Abstract

The Translational Science TS22 conference in Chicago in April 2022 was the first time post-pandemic that members of the Association of Clinical and Translational Science were able to meet up in person to share scientific advances. Given the remaining level of risk due to COVID-19, the meeting was designed as hybrid allowing virtual participation to some of the presentations. Prior to the meeting, JCTS Junior Editors were invited to report on the plenary sessions of the meeting. The present perspective constitutes a summary of three plenary sessions.

## Challenges to Translating Science to Action in a Public Health Emergency

Dr. Sonja Rasmussen, Professor in the Departments of Pediatrics and Epidemiology at the University of Florida College of Medicine and College of Public Health and Health Professions, delivered the plenary session titled “Challenges to Translating Science to Action in a Public Health Emergency.” Her presentation was based on her experiences of working with the Centers for Disease Control and Prevention (CDC) on various emergency responses, including the 2009 H1N1 influenza virus, the 2013 H7N9 influenza virus, the 2014 Middle East Respiratory Syndrome (MERS) coronavirus, the 2014 Ebola virus, and the 2016 Zika virus responses, as well as working on the response to COVID-19 in her role at the University of Florida. Dr. Rasmussen emphasized how working on responses to a public health emergency at CDC differed from day-to-day work at CDC: the decisions they had to make carried high stakes, involved a rapidly evolving situation, and were associated with limited available data and funding. She also underlined that in hindsight, it was easy to pinpoint mistakes and consider what could have been done better, but when a crisis arises, one must make the best possible decision with the information that is available at the time. It is important to act quickly despite uncertainty.

In the presentation, Dr. Rasmussen outlined three main lessons that her experience with emergency responses has taught her. These are: (1) preparedness is critical; (2) action is needed despite the lack of “perfect evidence”; and (3) effective communications are essential. One of the examples she used to demonstrate these lessons was the emergency response to the Zika virus. More specifically, she explained how there were various challenges associated with determining whether the virus caused birth defects. These challenges included that a large number of infected persons remained asymptomatic, that laboratory testing was not widely available, that there were no standardized case definitions of microcephaly, that no mosquito-borne viruses had been recognized as teratogenic in humans until that time, and that rumors were circulating about other possible causes such as insecticides. However, given the pressing need to protect pregnant women, Dr. Rasmussen and her team thoroughly reviewed the scientific literature and in a groundbreaking paper published in the *New England Journal of Medicine* [1] in 2016 they proposed that the available evidence demonstrated that Zika virus met Shepard’s criteria for teratogenicity. As a result, the CDC was able to strengthen its recommendations for pregnant persons to avoid traveling to high-risk areas.

Moreover, in this plenary session, Dr. Rasmussen outlined the public health decision-making process and explained four steps: the first step is to identify the problem. The second step is to review what is known, what is unknown, and potential assumptions. The third step is to list options to address the problem, and the fourth step is to determine the benefits and risks of each option. She also explained that some key factors that influence the timing and choice of public health interventions include the severity of the problem, the levels of scientific certainty of the findings, the extent to which the causes have been established, the operational and logistical feasibility, public and political perceptions, and legal considerations.

Furthermore, she discussed strategies for effective emergency and risk communication. She reviewed the six principles of Crisis and Emergency Risk Communication (CERC), namely, “Be First, Be Right, Be Credible, Express Empathy, Promote Action, and Show Respect.” She underlined that some important pitfalls to avoid are mixed messages from multiple experts, the release of information when it is too late, the adoption of paternalistic attitudes, and failure to counter rumors and myths in real time. She urged the audience that consistent messages are vital, and she underlined the importance of acknowledging uncertainty and not “over-reassuring.” In the future, when an emergency situation arises, she recommended acknowledging people’s fears, providing explanations, and offering specific things that people can do. Many of these issues are outlined in *The CDC Field Epidemiology Manual*, which can be downloaded at no cost on the CDC website here: https://www.cdc.gov/eis/field-epi-manual/index.html.

## Translating to Health Justice

The existing inequities in health and healthcare are multifactorial and it will take a comprehensive, collaborative approach to eliminate them. In this plenary session, Dr. Philip Alberti, Founding Director of the AAMC Center for Health Justice and Senior Director, Health Equity Research and Policy, unpacked the underlying meanings of health equity and health justice, framing them as population-level issues. He outlined the opportunities in advancing health equity and the role of clinical and translational science in promoting health justice, particularly at the population health (T4) level. He also highlighted the current advancements and resources available to serve as a framework for the clinical and translational science community as we tackle this complex and critical issue.

Inequity is multifaceted and must be addressed at the organization level (diversity, equity, and inclusion efforts), among different patient populations (healthcare equity) and within the larger community (healthy equity). These facets are intimately and intricately connected. However, the unifying root causes of the multiple facets of inequity are social, racial, economic, and other societal injustices. These are the same determinants of not only health inequity, but also of education, employment, and wealth inequity, and are woven into the fabric of our society via the political actions and inactions of those we elect to lead us. These root causes of inequity serve, at their core, to create unequal opportunities for certain populations in our country.

The CDC health impact pyramid outlines how typical healthcare interventions by professionals (clinical advice, counseling, etc.), while important, have a much smaller impact on overall health than do socioeconomic factors [2]. That is due to the multiple pathways in which the root causes of inequity impact socioeconomic factors for certain populations. As academic professionals, we must dig more deeply than simply optimizing healthcare when attempting to address health inequity. We must promote health justice by becoming part of a coordinated, aligned, multisectored set of interventions aimed at dismantling the societal structures that create and propagate inequity.

A key component of promoting health justice is community engagement. As was clearly outlined in the plenary session, “Translational Science and Community Health: Impact and Implications,” community engagement is a cornerstone of public health service and practice. The foundation of community engagement, and by extension a core component of health justice, is trust and trustworthiness [3]. Additionally, as clinical and translational scientists, part of earning community trust is to demonstrate a real commitment of time and financial resources to invest in truly T4-level translational research that translates health equity research into equity-driven changes in public health policies [4].

The onus is on healthcare organizations and health researchers to demonstrate their trustworthiness to the communities they are seeking to impact before any meaningful research endeavors within that community can be undertaken. Dr. Alberti provided an excellent framework via the AAMC Collaborative for Health Equity: Act Research Generate Evidence (CHARGE) initiative for meaningfully engaging community members not only as research participants, but as crucial and necessary members of the research development team, helping to guide the direction of future interventions. The resulting AAMC Principles of Trustworthiness (aamc.org/trustworthiness), which Dr. Alberti spearheaded, is a comprehensive tool that provides not only crucial insight into earning trust among communities but also a principle-based action plan for incorporating each principle into meaningful, community-engaged health equity research.

Ultimately, work towards health justice must first establish trustworthiness among the communities experiencing health inequities, must engage those communities in a meaningful exchange of ideas and information in a way that respects their collective knowledge, must ensure the focus is on creating health and healthcare opportunities, and must be intentional in incorporating research findings into actionable change in public health practices and policies at all levels.

## Translational Science and Community Health: Impact and Implications

Community engagement and collaboration are the cornerstones of public health science and practice. In this plenary session, the presenters outlined core principles of community engagement as it relates to translational science. Using real-world experiences, approaches to effective community engagement in translational research were discussed. Community-centered collaborations represent partnerships between academic institutions and the populations that they serve and are promoted by principles of trustworthiness, shared resources and decision-making, and health equity. Effective programs have a meaningful and sustainable impact on both institutions and the health of the community. Successful programs might also serve as a model for addressing current and future public health needs.

Dr. Linda Cottler, PhD, MPH, FACE, serving as this years’ Translational Science 2022 Program Committee Chair, and as Association of Clinical and Translational Science (ACTS) President-Elect, provided an opening welcome for the plenary session. Dr. Elizabeth Cohn provided an introduction to the session. Both Dr. Cottler and Dr. Cohn emphasized the relevance of community engagement in translational science and for the vision of the ACTS.

Dr. Sergio Aguilar-Gaxiola provided context to the session’s topic, answering the question: *why engage communities in a meaningful way?* Community collaboration is the foundation of successful public health practice with a shared goal of finding effective, long-term, and sustainable solutions for achieving health equity. Dr. Aguilar-Gaxiola described the *Assessing Community Engagement Conceptual Model* (ACE-CoM) program, for which he served as co-chair of the organizing committee. In ACE-CoM, community engagement remains the central focus with four principal surrounding domains: strengthened partnerships and alliances, expanded knowledge, improved health and health care programs and policies, and thriving communities. As part of the conceptual model, socioeconomic, racial, historical, and environmental drivers of health equity should be considered and addressed. As Dr. Aguilar-Gaxiola highlighted, ACE-CoM and other models of community engagement emerged as a key strategy for combating the COVID-19 pandemic, including the use of educational and vaccination programs. The success of such programs suggests its potential role in addressing future public health needs.

Dr. Elizabeth Cohn described the scientific basis for community engagement in translational research. Effective collaborations result in community empowerment, increased trust, a deeper understanding of cultural perspectives, strengthened science, and ultimately improved community health. A transition from a linear to a community-centered and dynamic model is needed. Using examples from two CTSAs (Weill Cornell and Columbia University), Dr. Cohn outlined the use of community advisory boards to build trust, promote community leaders, and identify community priorities. Pilot grant mechanisms serve as an important tool to support advocacy groups and community-based organizations and scientists, returning value to the community. Partnership and trust should be fostered at all phases of program development. When collaborations are begun, institutions and scientists should be knowledgeable about the community and be clear about their goals and purpose. In maintaining and sustaining successful partnerships, researchers should allow for community self-determination, releasing control over the priorities of the collaboration, and assuming a long-term commitment with the community.

Dr. Tabia Akintobi leads the Community Engagement program for the Georgia CTSA. The program aims to collaborate, disseminate and translate science, share resources, and build capacity and training opportunities centered around community health. Dr. Akintobi emphasized the need to *keep the end in mind*, where community partnerships are long-term and sustainable, regardless of institutional grant funding. Progress should be continually assessed and utilize metrics that measure outcomes that are important to the partnership. The Community Engagement program for the Georgia CTSA uses a Board Assessment Survey to track stakeholder satisfaction and leadership and participation metrics. More recently, the value of diversity and equity has been identified and will be serially measured. Dr. Akintobi highlighted lessons learned from their collaborations. Community governance, including having community members in leadership roles, is vital. Institutions should be “disease agnostic,” maintaining the ability to pivot energy and resources based on current priorities. Continuous assessment of the partnership allows for the early identification of potential obstacles.

Dr. Muredach Reilly provided experiences from the perspective of a CTSA Principal Investigator for Columbia University. Relationship building with the community should be based onn the transparency of goals and understanding the characteristics and priorities of the community. Dr. Reilly shared the vision and programs of the university Community Engagement Core Resource (CECR), which is supported by the CTSA. Program initiatives fall within three domains: community-based centers and support services for conducting research, capacity building through education, and CTSA integration and connection. The CECR has established several community-based health and wellness centers to provide direct health education and care and to improve access to meaningful translational research. Programs exist to train community health workers and to provide seed funding for cross-institutional and community collaborations focused on translational research and community health. Recent actions supported by the CTSA include the establishment of a task force addressing structural racism in clinical trials. The importance of diversity and a team science approach for successful community engagement was emphasized.

## Summary

The plenary sessions highlighted three areas of significant importance and relevance for clinical and translational science. Dr. Rasmussen gave an informed perspective of society response to pandemics and the considerable uncertainties facing public and government agencies. She underscored the importance of a strategic communication given the dynamic level of knowledge and gave the audience a flair of the challenges facing these institutions.

Dr. Alberti discussed the role of clinical and translational science in promoting health justice, particularly at the population health (T4) level and some key takeaways from his presentation were as follows:

- Health equity is the goal. Health justice is the path.
- The fundamental causes of inequity are social, racial, and economic injustice.
- Measuring healthy equity is not the same as measuring health inequities. In healthy equity work, the focus should be on creating equal opportunities rather than on mandating equal outcomes.
- To achieve health equity, medicine and research must be the best partners they can be in the multisector collaborative necessary to change underlying structures and systems.
- Organizations of all levels (private, local, state, federal) must ensure the commitment to health justice research is just as strong as the commitment to research in biological and other sciences.

The four speakers addressing translational science and community health provided a comprehensive insight and outlined core principles of community engagement as it relates to translational science. Some key points from their presentation were:

- Community engagement and collaboration are the cornerstones of public health science.
- Partnerships are promoted by principles of trustworthiness, shared resources and decision-making, and health equity.
- Programs should be transparent with community members regarding the purpose and goals of the partnership.
- Successful programs support both community-based organizations and researchers with the primary goal of improving community health.
- The progress of a community engagement program should be continually assessed.

In addition to the plenary sessions, a number of oral and poster presentations were given, representing the broad spectrum of clinical and translational research. We now look forward to an equally exciting ACTS Translational Science 23 meeting.
