# Translational Science 22 conference proceedings – CORRIGENDUM

**DOI:** 10.1017/cts.2022.477

**Published:** 2023-03-14

**Authors:** Elina E. Pliakos, Laneisha K. Tague, Roy A. Poblete

The above article [1] published with the incorrect middle initial for Roy Poblete. The author’s correct full name is Roy A. Poblete.

The original article has been corrected online to rectify this error.
